# Novel mutation of the *PRNP *gene of a clinical CJD case

**DOI:** 10.1186/1471-2334-6-169

**Published:** 2006-11-27

**Authors:** Konstantia Kotta, Ioannis Paspaltsis, Sevasti Bostantjopoulou, Helen Latsoudis, Andreas Plaitakis, Dimitrios Kazis, John Collinge, Theodoros Sklaviadis

**Affiliations:** 1 Prion Disease Group, Laboratory of Pharmacology, School of Pharmacy, Aristotle University of Thessaloniki, Thessaloniki, Greece; 2 Neurological Clinic, General University Hospital G. Papanicolaou, Exohi, Thessaloniki, Greece; 3 Department of Neurology, School of Health Sciences, University of Crete, Heraklion, Crete, Greece; 4 MRC Prion Unit and Department of Neurodegenerative Diseases, Institute of Neurology, University College London, Queen Square, London WC1N 3BG, UK

## Abstract

**Background:**

Transmissible spongiform encephalopathies (TSEs), a group of neurodegenerative diseases, are thought to be caused by an abnormal isoform of a naturally occurring protein known as cellular prion protein, PrP^C^. The abnormal form of prion protein, PrP^Sc ^accumulates in the brain of affected individuals. Both isoforms are encoded by the same prion protein gene (*PRNP*), and the structural changes occur post-translationally. Certain mutations in the *PRNP *gene result in genetic TSEs or increased susceptibility to TSEs.

**Case presentation:**

A 70 year old woman was admitted to the hospital with severe confusion and inability to walk. Relatives recognized memory loss, gait and behavioral disturbances over a six month period prior to hospitalization. Neurological examination revealed Creutzfeldt-Jakob disease (CJD) related symptoms such as incontinence, Babinski sign and myoclonus. EEG showed periodic sharp waves typical of sporadic CJD and cerebrospinal fluid analysis (CSF) was positive for the presence of the 14-3-3-protein. As the disease progressed the patient developed akinetic mutism and died in the tenth month after onset of the disease symptoms. Unfortunately, no autopsy material was available. *PRNP *sequencing showed the occurrence of a point mutation on one allele at codon 193, which is altered from ACC, coding for a threonine, to ATC, encoding an isoleucine (T193I).

**Conclusion:**

Here we report a novel mutation of the *PRNP *gene found in an elderly female patient resulting in heterozygosity for isoleucine and threonine at codon 193, in which normally homozygosity for threonine is expected (T193). The patient presented typical clinical symptoms of CJD. EEG findings and the presence of the 14-3-3 protein in the CSF, contributed to CJD diagnosis, allowing the classification of this case as a probable CJD according to the World Health Organization (WHO) accepted criteria.

## Background

Transmissible spongiform encephalopathies (TSEs) are a group of neurodegenerative diseases of the central nervous system. These diseases share common features and can be found in both humans and animals. The infectious agent of TSEs is believed to be an abnormal isoform of a naturally occurring protein known as cellular prion protein, PrP^C ^[1]. The abnormal prion protein isoform (PrP^Sc^) accumulates in the brains of affected individuals. Both isoforms are encoded by the same gene *PRNP*, and the structural changes occur post-translationally. The lack of the endogenous PrP protein prevents the development of TSEs, as has been demonstrated in transgenic mice lacking the *PRNP *gene [2]. On the other hand, mutations that exist in the *PRNP *gene might facilitate the development of TSEs [3-5]. The most common TSE in humans is Creutzfeldt-Jakob disease (CJD), accounting for 85% of all TSE cases in humans [6]. There are three types of CJD, sporadic CJD (sCJD), infectious CJD (iCJD) and genetic CJD (gCJD). sCJD arises spontaneously, with unknown aetiology and affects mainly the elderly population at a frequency of one per million per year. iCJD is caused by the consumption of prion contaminated food or by medical treatments with prion contaminated biological materials and surgical instruments [7]. In gCJD, mutations in the *PRNP *gene predispose to disease [7,8] by causing the expression of PrP protein with modified primary structure.

Several mutations have been identified in the prion protein, encoded by the *PRNP *gene. More than thirty have been associated or directly linked to TSEs [9-11]. Here we report a novel mutation in codon 193 (T193I) of the *PRNP *gene, in a patient with clinical features and EEG findings typical of CJD. Normally, codon 193, comprising nucleotides ACC, codes for a threonine, whereas in the case reported here it has been changed to ATC, thus encoding an isoleucine.

## Case presentation

### Patient

The patient was a 70 year old woman, with a history of diabetes mellitus, who was admitted to the hospital with confusion and inability to walk. Six months prior to admission, her relatives had started recognizing neurological symptoms, such as gait disturbance, memory loss and behavioral disturbances (irritability and aggression). Over the six month period that preceded the admission to the hospital, a rapid deterioration of her cognitive function was observed. She also displayed visual hallucinations and delusions requiring antipsychotic treatment.

On admission, the patient was bedridden, incontinent, severely confused with generalized hyperreflexia, bilateral Babinski sign, startle responses elicited by mild external stimuli, and multifocal generalized myoclonus which evolved to rhythmic. Two months later she developed akinetic mutism. The patient died three months after hospitalization.

**Figure 1:**
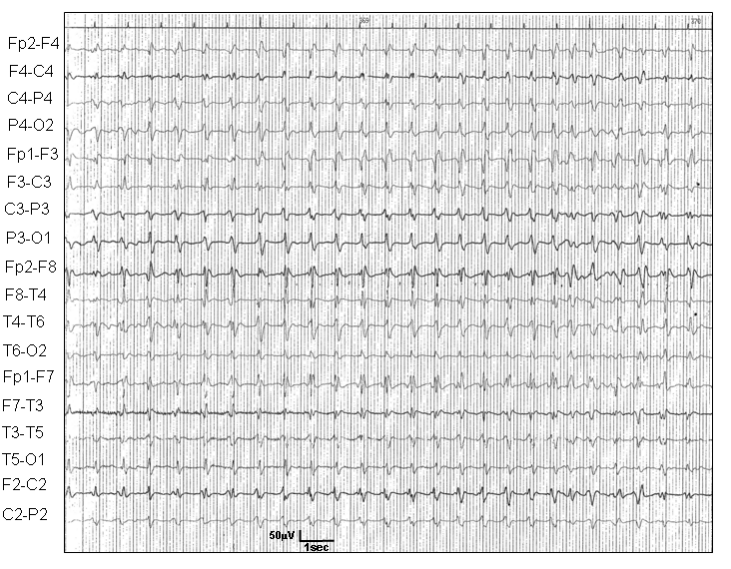
Electroencephalogram of the suspected CJD patient showing typical periodic bursts of triphasic sharp waves.

Routine hematological and biochemical examinations produced normal results. CSF examination (protein, glucose and cell count) was normal, while EEG showed generalized findings, periodic triphasic sharp waves, recurring at intervals of 0.5–1 sec (Fig 1). Magnetic resonance imaging of the brain showed no significant abnormalities, except for a minor brain atrophy which could be related to the patient's age.

There was a lack of family history concerning neurological disorders. Although the clinical course followed the typical rapid progression of CJD, the relatives did not consent to having a neuropathological examination carried out, neither did they agree to further genetic investigation of the immediate family members, and thus it has not been possible to determine if the mutation has been passed to the patient's offspring.

### 14-3-3 protein detection in the CSF

Cerebrospinal fluid (CSF) was collected by lumbar puncture. Both the lumbar puncture and the EEG, were performed three and eight days after the patient's admission to the hospital (about six months after the occurrence of the reported symptoms observed by family members).

The proteins contained in 30 μl of CSF were separated on a 12% polyacrylamide gel and then transferred onto a polyvinylidene fluoride (PVDF) membrane. The membrane was stained as described [12] with a polyclonal anti-14-3-3β antibody (Santa Cruz, sc-629) and an alkaline phosphatase conjugated secondary antibody (Pierce, 31340). CDP-Phototope Star (New England Biolabs, N701) was used to develop the membrane.

**Figure 2:**
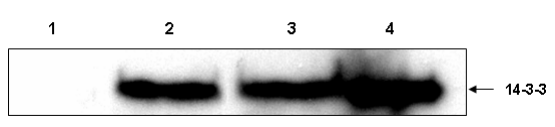
Immunoblot detection of 14-3-3 in the CSF. The patient was tested positive for the presence of 14-3-3 in the CSF. Lane 1: healthy control, lane 2: patient sample, lane 3: positive control (CSF) and lane 4: positive control (brain tissue homogenate)

Detection of 14-3-3 protein in CSF, is considered to be a sensitive and specific marker for sporadic CJD. CSF tested positive twice for the presence of 14-3-3 protein (Fig. 2), supporting a probable CJD diagnosis [13,14].

### DNA purification and sequencing

Genomic DNA was isolated from whole blood using Promega's Wizard genomic DNA purification kit according to the manufacturer's instructions. The proper quality and quantity of the genomic DNA for polymerase chain reaction (PCR) amplification was confirmed by spectrophotometry. The *PRNP *open reading frame was amplified by PCR with the following primers designed as cloning primers; HumoS: 5'- *GCT CTA GAG *C**AT GGC GAA CCT T **and HumoAS: 5'- *GCT CTA GA***T CAT CCC ACG ATC AGG AA**. The bases of the primers corresponding to the open reading frame of *PRNP *are shown in bold. Amplification was confirmed by agarose gel electrophoresis. Sequencing was performed first at MWG-Biotech (Ebersberg, Germany) and then the sequencing results were confirmed by two other independent laboratories (Department of Neurology, University of Crete, MRC Prion Unit and Department of Neurodegenerative Diseases, University College, London).

**Figure 3:**
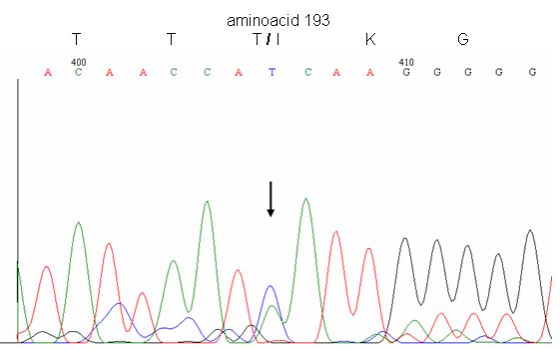
Electropherogram of the DNA sequencing showing the position 193 were the mutation was detected. Codon ACC codes for a Threonine and codon ATC for an Isoleucine. The arrow indicates the position on the electropherogram, where both C and T are present.

*PRNP *sequence analysis revealed the existence of a causative mutation at codon 193. Upon comparison with the 762 bp sequence of the human PrP cDNA record (Genebank accession number M13899), it was found that the sequenced *PRNP *gene exhibits a point mutation at codon 193, comprising nucleotides 577–579. The point mutation occurs as a C to T transition at position 578 which causes the switch of codon 193 from ACC, encoding threonine, to ATC, encoding isoleucine. As shown on the electropherogram (Fig. 3), the patient is heterozygous for threonine and isoleucine at codon 193. The patient was found to be homozygous for methionine at codon 129 (M129).

## Conclusion and discussion

The genotype of the host seems to play an important role in prion disease pathogenesis. Susceptibility to prion diseases is enhanced if the individual possesses certain genotypes. The human genetic TSEs account for 5–15% of human TSE cases and have been linked to specific mutations of the *PRNP *gene [9,10,15]. Susceptibility to sCJD has been associated with homozygosity for methionine at the polymorphic codon 129 [16,17]. In the Greek population 50% homozygosity for M129 has been reported [18].

Here we report the case of a 70 year old female patient carrying the T193I mutation, who presented typical clinical symptoms of CJD. The presence of a mutation in the *PRNP *gene along with the presentation of symptoms typical for CJD supports the diagnosis of probable CJD according to the WHO accepted criteria [13]. The observed mutation was associated with homozygosity for methionine at codon 129. The patient's age at onset of the symptoms and the homozygosity M129 is consistent with the average age at onset observed in other gCJD cases [9].

The PrP protein primary structure is well conserved over a variety of mammals [19]. Interestingly, T193 of the human PrP protein can be found in the homologue proteins at the same position in all thirty six species listed in the cited publication, except in shrew, in which it is replaced by serine. Threonine and serine, are both hydrophilic polar amino acids with uncharged side chains, while isoleucine is a hydrophobic amino acid. The T193I mutation reported here, may affect the three dimensional structure of the protein. Since TSEs are linked to conformational changes of the prion protein, this mutation may be associated with the occurrence of the disease.

The mutation reported here results in an amino acid change within α-helix 2 (H_2_) of the PrP protein, which comprises amino acids 173–194 [20]. H_2 _is characterized by the occurrence of different causative mutations, most of which are linked to the appearance of a CJD phenotype [10].

Of particular interest is the T188A mutation, which is the most closely located mutation to codon 193 on H_2 _that has been reported [21]. Similarly to T193I, in the T188A case, threonine is replaced by a hydrophobic residue and both cases emerged with homozygosity for methionine at codon 129. Both patients were women of similar age i.e. 70 and 69 years old, respectively, and the brain MRI scan revealed no typical CJD findings, but a minor brain atrophy. EEG and the CSF analysis for the 14-3-3 protein were typical for sCJD in both cases. Clinical symptoms like the failure of cognitive abilities, myoclonus and visual disturbances were observed in both patients. Additionally, both patients share the absence of family history for dementia, which is probably caused by the reduced penetrance of the mutation [9,22].

Codon 196 is very closely located to codon 193 although it does not belong to H_2_. The E196K mutation associated with M129 homozygosity [22] affected a 69 year old woman, who in contrast to the patient reported here, had a family history of dementia. The onset symptoms in both patients included behavioral abnormalities, albeit of a different type (the patient carrying the E196K mutation displayed emotional lability, inappropriate giggling, anorexia versus irritability and aggression). Gait disturbances were observed in both patients and both became bedridden as the disease progressed. Furthermore, both patients tested positive for the presence of 14-3-3 protein in the CSF. However, the EEG of the patient carrying the E196K mutation showed no periodic activity and there was a slight difference in the period from the onset of symptoms to death (10 versus 12 months).

During the CJD surveillance program supported by the Greek Ministry of Health, a total of twenty five patients suspected of having CJD, and ten of their relatives, were genotypically examined with respect to the *PRNP *gene. Full sequencing of the above samples revealed no abnormalities in the *PRNP *sequence and in all samples homozygosity for threonine at codon 193 was found. These data indicate that the T193I alteration of the PrP protein reported here is a mutation rather than a rare polymorphism.

Such mutations may either be inherited by the individual from the parents following the rules of Mendelian inheritance, or they may occur spontaneously. In the second case, a lack of family history should be expected. Lack of family history in gCJD cases is often reported [9] and it is suggested to occur due to the partial penetrance of the mutation.

## Competing interests

The author(s) declare that they have no competing interests.

## Authors' contributions

KK conducted the immunoblot analysis and drafted the manuscript. IP conducted the immunoblot analysis, the PCR amplification and drafted the manuscript. SB and DK performed the clinical observations and reviewed the manuscript. HL and AP performed the sequencing reaction and reviewed the manuscript. JC repeated and confirm the sequencing analysis and TS overviewed the work and reviewed the manuscript. All authors read and approved the final manuscript.

## Pre-publication history

The pre-publication history for this paper can be accessed here:


